# Medicine

**Published:** 1905-03

**Authors:** Theodore Fisher


### Progress of the Medical Sciences.

### MEDICINE.

**Pericarditis and pericardial effusion** are the subject of several papers. One upon pericarditis commences with the following remark: "Pericarditis is, as a rule, more damaging to the heart than endocarditis. It is almost certain to shorten the life of the person afflicted unless an intercurrent disease should anticipate its effect."[1] It is not perhaps the function of a review such as this to criticise, yet it is difficult to refrain from expressing

[1] Riesman, *Am. J. M. Sc.*, 1904, cxxviii. 466.

some measure of dissent from this remark. In the first place, a distinction needs to be drawn between pericarditis due to rheumatism and to other causes; in the second place, the age at which a patient is attacked is extremely important. Having paid attention to this subject, we believe that the remark about the serious nature of pericarditis certainly applies to pericarditis associated with rheumatism when it occurs before the age of twelve years. In the great majority of cases it is then followed by early death. Pericarditis of rheumatic origin arising also between the ages of twelve and twenty may be followed by serious consequences; but after the age of twenty, if recovery from the acute attack takes place, it is only in very exceptional cases that any ill-effects subsequently follow.

**With regard to other forms of pericarditis,** whether occurring in childhood or adult life, complete recovery is the rule, and any curtailment of life which can be attributed to adhesion of the pericardium is the exception. In adult life one of the rarest morbid conditions to be seen on the *post-mortem* table is adhesion of the pericardium which has occasioned cardiac failure.

In the above paper there are many other statements upon which comment could be made, but it is much more easy to criticise than to furnish guiding principles. The subjects of the diagnosis of pericardial effusion or of general adhesion of the pericardium, with which the paper largely deals, are of great difficulty. This paper summarises the various physical signs which are thought to provide means for diagnosis, but it cannot be said that it adds anything to our present knowledge. The paper has been referred to because it lies between two others that afford, perhaps, more practical points for consideration.

The article[1] which precedes it deals with **pyo-pericarditis**. The writers upon this subject lay stress upon the well-known fact, apt sometimes to be forgotten, that purulent pericarditis is a frequent complication of pneumonia. They mention also what is also well known, but is perhaps not so important, that it is associated with local and general septic conditions. These writers consider that with the onset of pericarditis the temperature in pneumonia is likely to be lowered rather than raised above its former level, that pain over the heart is frequently present, and that suspicion of the presence of pericarditis should always be aroused when the dyspnœa is greater than the physical signs in the lungs would suggest. It is needless, however, to remark that sero-fibrinous pericarditis is considerably more common in pneumonia than the purulent variety, and that only in a small number of this variety is the effusion present in sufficient quantity to justify surgical interference.

[1] Scott and Le Conte, *Ibid.*, p. 447.

The presence of this effusion is detected by careful percussion. Three cases of pericarditis associated with pneumonia are reported by these writers, and another of septic origin, in which the pericardium was aspirated, but the methods of surgical procedure perhaps need not be referred to until other papers upon this subject have been briefly noticed.

A paper upon[1] **paracentesis of two cases of pericarditis** follows that of Dr. Riesman, under whose care both of the patients were. These cases illustrate well the difficulty of diagnosis of moderate degrees of pericardial effusion. They were both under the care of a physician interested in this subject, as his paper, which deals fully with the signs of pericardial effusion shows, yet Dr. Roberts, who was called in to aspirate and record the cases, obtained only three ounces of fluid in one case and none at all in the other. In this case the absence of fluid was proved *post mortem*. Two cases are, however, recorded by Thayer[2], where the diagnosis was far less difficult on account of the large amount of effusion present. Both cases were in male adults—one aged 26 and the other aged 56. In both the effusion was tuberculous. The præcordial dulness in the first case extended from the first rib above outwards to the nipple line on the right side and on the left to the posterior axillary fold. The heart-sounds were almost absent, and when they could be heard best "so extremely feeble and distant that nothing definite could be determined as to their character." Here in passing it may be remarked that in a case I saw, where pericardial effusion was so great that it was thought to be fluid in the left pleural cavity, and only found to be in the pericardium after death, the heart-sounds were unusually sharp and loud, so loud in fact that their peculiar intensity was commented upon in the clinical record. But to return to the case recorded by Thayer, the decision was arrived at that aspiration should be performed and 1,250 c.c. of clear fluid were withdrawn. In the second case, although the area of dulness was large the aspiration failed to withdraw fluid. After death, however, 1,200 c.c. of fluid were found in the pericardium. Although the heart was not fixed by adhesions it lay in front of the effusion, and the aspirating needle had come into contact with the heart-wall. A case of some interest in this connection is recorded by Dabert.[3] Aspiration of the pericardium was attempted in a lad aged 17. When lying in bed no fluid could be obtained, but when raised to the sitting posture 10½ ounces were withdrawn. When the patient was recumbent the heart could be felt in contact with the needle. This was a case of rheumatic pericarditis, and the patient, although considered in an almost

[1] J. B. Roberts, *Ibid.*, p. 473. [2] *Johns Hopkins Hosp. Bull.*, 1904, xv. 149.

[3] *Berl. klin. Wchnschr.*, quoted in *Med. Rev.*, 1905, viii. 31.

hopeless condition when the paracentesis was performed, recovered.

If recovery can be attributed to the withdrawal of comparatively so small a quantity of fluid, it does not seem probable that the removal of pressure is mainly responsible for the relief of the heart. It is more probable that the removal of deleterious toxins is the main beneficial factor. At least, it is remarkable how rapidly the pericardium will yield under pressure. Thayer refers to recorded cases in which $4\frac{1}{2}$ lb. and $5\frac{1}{2}$ lb. of blood have been removed from the pericardium in cases of scurvy with termination in recovery. It may be mentioned in passing that this distensibility of the pericardium, under circumstances apart from that of acute pericardial inflammation, shows the fallacy of the view which attributes enlargement of the heart after rheumatic pericarditis to failure of the weakened pericardium to support the heart. Besides, curiously enough, in the most acute forms of pericarditis, suppurative pericarditis, where it would be expected that the pericardium should yield most readily, there is no dilatation of the heart. This, however, is wandering from the subject, yet it is well to bear in mind that although dilatation of the heart in rheumatic pericarditis cannot, it seems to me, be attributed to weakening of the pericardium, the presence of such dilatation of the heart in pericarditis from the point of view of diagnosis is important. The dilatation may be very rapid in cases of rheumatic pericarditis, and in the great majority of instances where fluid in considerable quantity is thought to be present there is virtually nothing but very marked dilatation of the heart.

A brief reference to **methods of procedure in paracentesis of the pericardium** is necessary. Difference of opinion has existed as to which is the best site for puncture. The dangers to be avoided are: (1) infection of the pleura, (2) injury to the internal mammary vessels, (3) puncture of the heart. Apparently wherever a puncture is made it is almost impossible to avoid wounding the pleura, so that after considering various points in connection with the above, Thayer comes to the conclusion that the sixth space about the mamillary line is the best spot for exploratory puncture or aspiration, provided there is no evidence that the heart extends so far out. Should the apex reach that point, then it will be necessary to explore further to the left. Roberts, in the above-mentioned cases, appears to have chosen this spot. Scott and Le Conte, speaking of incision of the pericardium for purulent pericarditis, select the fourth left interspace, beginning one inch from the sternal border and extending to the normal position of the apex-beat of the heart. Pendlebury,[1] however, advises a

[1] *Lancet*, 1904, ii. 1145.

vertical incision apparently not far from the left border of the sternum, but the exact situation in his paper is not given, although other details of two cases operated upon are described. In one portions of the sixth and seventh costal cartilages were removed, and in another a portion of the seventh cartilage.

THEODORE FISHER.

### SURGERY.

